# Peripheral nerve blocks of wrist and finger flexors can increase hand opening in chronic hemiparetic stroke

**DOI:** 10.3389/fneur.2024.1284780

**Published:** 2024-02-21

**Authors:** Hendrik A. Dewald, Jun Yao, Julius P. A. Dewald, Antoun Nader, Robert F. Kirsch

**Affiliations:** ^1^Department of Biomedical Engineering, Case Western Reserve University, Cleveland, OH, United States; ^2^Department of Physical Therapy and Human Movement Sciences, Northwestern University, Chicago, IL, United States; ^3^Department of Biomedical Engineering, Northwestern University, Evanston, IL, United States; ^4^Department of Anesthesiology, Northwestern University, Chicago, IL, United States; ^5^Cleveland FES Center, Louis Stokes Cleveland Veterans Affairs Medical Center, Cleveland, OH, United States

**Keywords:** nerve block, stroke, anesthesia, hand opening, grasp, FES, upper extremity synergies, paresis

## Abstract

**Introduction:**

Hand opening is reduced by abnormal wrist and finger flexor activity in many individuals with stroke. This flexor activity also limits hand opening produced by functional electrical stimulation (FES) of finger and wrist extensor muscles. Recent advances in electrical nerve block technologies have the potential to mitigate this abnormal flexor behavior, but the actual impact of nerve block on hand opening in stroke has not yet been investigated.

**Methods:**

In this study, we applied the local anesthetic ropivacaine to the median and ulnar nerve to induce a complete motor block in 9 individuals with stroke and observed the impact of this block on hand opening as measured by hand pentagonal area. *Volitional* hand opening and *FES*-driven hand opening were measured, both while the arm was fully supported on a haptic table (*Unloaded*) and while lifting against gravity (*Loaded*). Linear mixed effect regression (LMER) modeling was used to determine the effect of *Block*.

**Results:**

The ropivacaine block allowed increased hand opening, both volitional and FES-driven, and for both unloaded and loaded conditions. Notably, only the *FES*-driven and *Loaded* condition’s improvement in hand opening with the block was statistically significant. Hand opening in the *FES* and *Loaded* condition improved following nerve block by nearly 20%.

**Conclusion:**

Our results suggest that many individuals with stroke would see improved hand-opening with wrist and finger flexor activity curtailed by nerve block, especially when FES is used to drive the typically paretic finger and wrist extensor muscles. Such a nerve block (potentially produced by aforementioned emerging electrical nerve block technologies) could thus significantly address prior observed shortcomings of FES interventions for individuals with stroke.

## 1 Introduction

An estimated 9.4 million Americans 20 years of age or older self-reported having had a stroke, with projections suggesting that an additional 3.4 million Americans may join them by 2030 (1). Moderately impaired individuals have a reduced ability to open their hands, while severely impaired individuals are often unable to open their impaired hand at all—especially due to involuntary flexion forces at wrist and fingers linked to increasing abduction load at the shoulder (2) and flexor hypertonia (3). While motor impairments at the paretic hand are due to multiple factors (4), of particular importance are *overactive wrist and finger flexors* and simultaneous *extensor weakness* (5). In particular, the proportional reduction of hand opening in relation to shoulder abduction loading results largely from the expression of the “*flexion synergy*” (i.e., abnormal coupling between shoulder abduction and elbow/wrist and finger flexion) (6–8), thought to be due to greater reliance on reticulospinal projections following a hemiparetic stroke (9). Furthermore, the flexor hypertonia may be related to the possible upregulation of monoaminergic coeruleospinal projections (10). The presence of hyperactive stretch reflexes, in comparison, may not play a major role in stroke disability (5) compared to the expression of said flexion synergy (11). As passive muscle properties are also largely unchanged (4, 12), it is likely the overactive wrist and finger flexors (*particularly* the flexion synergy) and extensor weakness that limits hand use in stroke.

The reduction in hand opening while lifting, induced by said flexion synergy expression (2), persists even when assisted by functional electrical stimulation (FES), limiting the effectiveness of FES interventions (13–15). Limited ability to open one’s hand can also lead to the “learned disuse” of the whole paretic arm (16), potentially worsening patient outcomes over time. Without the useful end-effector necessary for many activities of daily living, the impact of reach-focused rehabilitation interventions (17, 18) can be reduced as well.

A possible method for improving hand opening during lifting is thus to inhibit the “over-activated” flexors. One of the most commonly utilized clinical methods for reducing hyperactive flexor activity is the use of botulinum toxin A, which temporarily reduces function at the neuromuscular junction (19, 20). This approach has been employed with initially encouraging results (21–23). However, the approach also has a number of limitations. While the “therapeutic effect” is reported to last 3 months, the magnitude of that effect varies significantly within this window with peak effect occurring around 5 weeks and gradual reduction of effect thereafter (24). Therefore, most patients require repeat injections, often in combination with physical therapies, every 3 to 4 months (25). A review article has shown strong evidence that botulinum toxins reduce hypertonia and spasticity (i.e., hyperactive stretch reflex), but its effect on improving hand and arm function is less compelling (26). Botulinum toxins further reduce the strength of the already paretic muscle, which may negatively impact function (27). Some individuals even develop neutralizing antibodies to the toxin, rendering the intervention ineffective with repeat injections (28). Finally, evidence also indicates potential long-term concerns related to increased muscle passive stiffness, possibly due to muscle extracellular matrix proliferation (12, 29–32). An alternative worth exploring is the use of FES-based methods that provide instantaneous, controllable, and reversible blocking of peripheral nerve transmission (33–35). However, these methods are still under development, and their feasibility in improving voluntary hand opening and/or FES-driven hand opening, with or without arm lifting, has yet to be evaluated.

In this study we temporarily relaxed the finger flexor muscles using an anesthesia block of the median and ulnar nerves as a proxy for future electrical block techniques. Specifically, ropivacaine was selected for perineural injection into both median and ulnar nerves to provide an adequate motor block duration (~8.7 h) (36, 37) with low required dosages (5 mL) (38). The efficacy of such a temporary nerve block approach was then assessed by measuring improvements in volitional- and FES-assisted hand opening after the application of the anesthesia nerve block, both when the arm was in a relaxed state and when participants had to raise their paretic arm against gravity by abducting at the shoulder.

## 2 Materials and methods

### 2.1 Participants

Having conducted a power analysis based on earlier hand opening data involving stroke participants (2), in which we assumed a nerve block effect size of a 20% increase in hand opening and similar variance, we enrolled a total of 10 individuals with chronic stroke (occurring more than 1 year ago) for this proof-of-concept study. The respective demographics of these participants are detailed in Table 1. Participants were recruited from the Shirley Ryan AbilityLab/Physical Therapy and Human Movement Sciences Clinical Research Registry and from the greater Chicago area. Other main inclusion/exclusion criteria included: (1) paresis confined to one side, with an ability to lift the arm up to the horizontal plane while maintaining 90 degrees elbow flexion; (2) no allergies to lidocaine or ropivacaine, and no use of contraindicated medications; (3) no recent or prior long-term use of other chemodenervation approaches, such as botulinum toxin, in the hand and wrist flexor muscles; (4) absence of any severe concurrent medical problems (such as cardiorespiratory impairment) or any acute/chronic pain conditions in the upper extremities or spine greater than 5 on the 10-point visual analog scale; (5) no use of a cardiac pacemaker, implanted defibrillator, neurostimulation device, or similar implanted electrical equipment.

**Table 1:** Participant demographic and clinical data.

ID	FMA UE (/66)	Impaired arm	Dominant hand pre-stroke	Age	Sex	Years post stroke
S01	33	L	R	57	M	24
S02	18	R	R	64	M	12
S03	29	L	L	68	M	13
S04	24	R	R	41	M	7
S05	20	L	L	63	M	15
S06	20	R	R	74	F	18
S07	43	L	R	67	M	11
S08	47	L	R	61	M	7
S09	13	L	R	73	F	30
S10	47	L	R	52	M	12

FMA UE: Fugl–Meyer assessment of the upper extremity, a measure of impairment where the maximum score of 66 suggests a level of ability indistinguishable from able-bodied individuals. L, Left; R, right; M, male; F, female.

All participants gave written informed consent for participation in this study and the publication of any potentially identifiable images or data included in this article, as approved by the Northwestern University Institutional Review Board (IRB #STU00213403).

### 2.2 Experimental setup

The experiment was performed on the Arm Coordination Training 3D (ACT^3D^) system (39, 40), which consists of a modified HapticMaster robot (Moog-FCR BV, the Netherlands) and a Biodex chair and T-Base support system (Biodex Medical Systems, Shirley, NY). The ACT^3D^ was used to measure arm configuration and modulate shoulder abduction load. Under the “*Unloaded*” condition, a frictionless virtual haptic table was provided by the ACT^3D^, and under the “*Loaded*” condition, a shoulder abduction load of 100% of the participant’s limb weight (41) was imposed.

**Figure 1:**
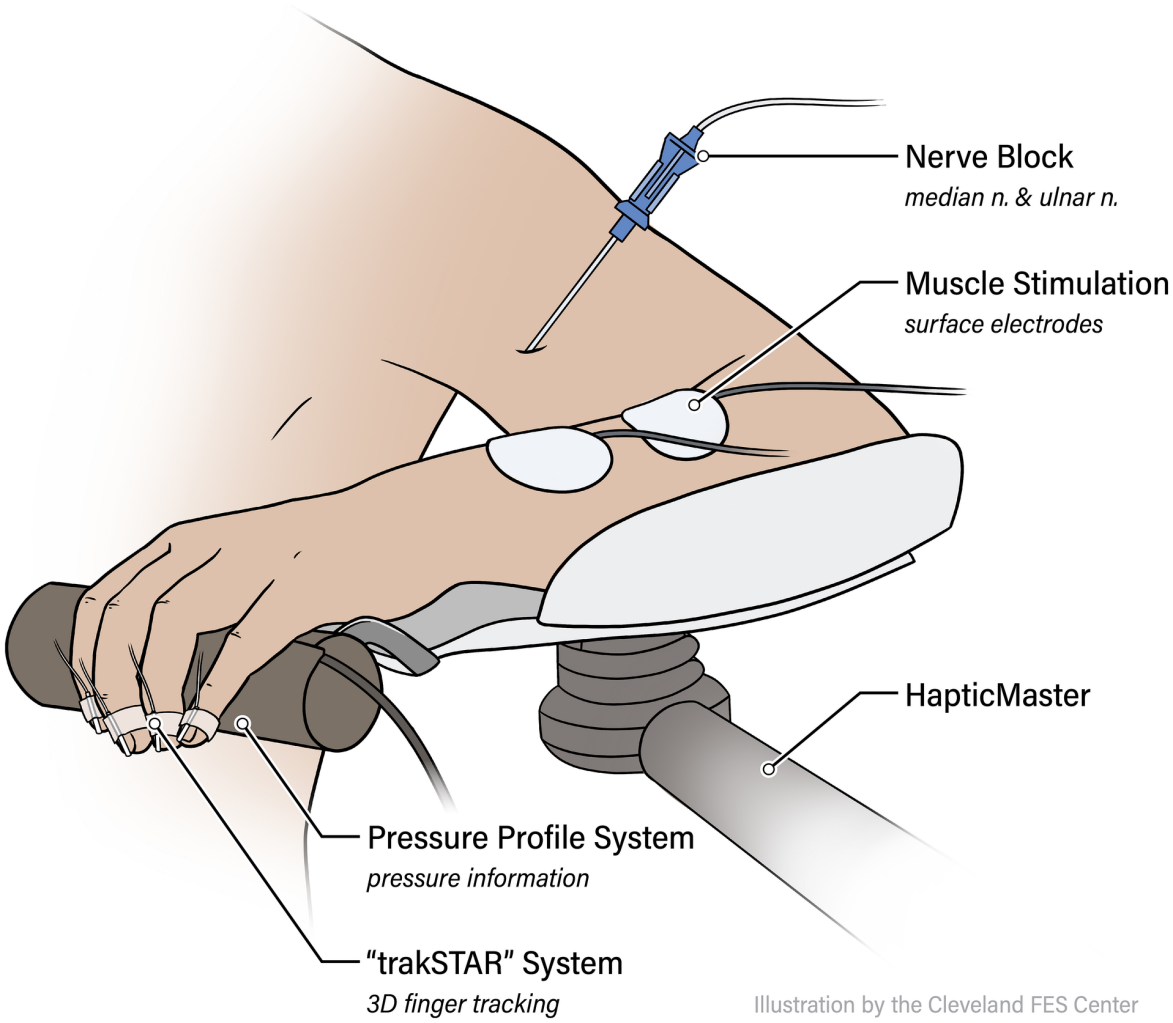
Experimental setup. Participants are attached via an orthosis to the ACT^3D^ assistive/loading device, and instrumented with the trakSTAR position sensors and PPS pressure mat. Functional electrical stimulation (FES) electrodes are placed on forearm flexors (flexor digitorum superficialis, or FDS) and extensors (extensor digitorum communis, or EDC).

Participants were seated in the Biodex chair with the trunk and shoulder strapped securely to prevent compensatory movements. Following a short series of wrist and finger stretches, the participant’s impaired arm was attached to the forearm orthosis of the ACT^3D^ and placed in a “Home Position” of 85° shoulder abduction (SABD), 90° elbow flexion (EF), 40° shoulder flexion (SF), and 0° wrist extension (WE) (see Figure 1). The participant’s fingers, thumb, and palm were placed around a cylinder attached to the distal end of the forearm orthosis. On the cylinder, a pressure sensor mat (Custom TactArray Sensing System, Pressure Profile Systems Inc., Los Angeles, CA) was mounted circumferentially. This pressure mat contained 27 by 21 sensors across a 6.4 by 5.1-inch surface area, with each sensor able to record up to 50 PSI with a pressure sensitivity of 0.15%. Furthermore, five Model 180 sensors from two linked trakSTAR systems (Northern Digital Company, Waterloo, ON, Canada) were placed on each of the tips of the 5 fingers to record the hand aperture to an accuracy of 1.4 mm Root Mean Square Error.

#### 2.2.1 Assistive functional electrical stimulation parameters

A 2-channel E-Wave surface stimulator (Zynex Medical, Englewood, CO) was used to stimulate forearm compartment finger muscles, with one set of bipolar cutaneous electrodes placed over the flexor digitorum superficialis (FDS), and the other bipolar electrode pair placed over the extensor digitorum communis (EDC). The E-Wave was set up with a 200 μs pulse width duration and a 28 Hz biphasic stimulation paradigm that balances participant comfort with minimization of fatigue (42, 43). For each participant, each channel’s appropriate intensity was found by incrementally increasing the current amplitude until a visible plateau of effect was reached, or the participant expressed discomfort.

### 2.3 Protocol

#### 2.3.1 Before anesthesia (*Unblocked*)

After the instrumentation setup, each participant performed a series of hand *Opening* and *Closing* tasks. These tasks were done as groups of at least 3 repetitions per set of conditions, under the following 2-by-2 conditions, themselves selected in random order: shoulder abduction loading condition (*Loaded* vs. *Unloaded*) and driving condition (*Volitionally* vs. *FES* driven).

All tasks were performed with the tested arm/hand at the “Home Position.” An auditory cue, 200 ms after the start of data collection, was used to trigger the participant to start the required task. Under the *Unloaded* condition, the participant opened or closed their hand volitionally (if a *Volitional* trial) or simply relaxed to let FES drive the task (if *FES* trial) for 6 s with the arm resting on the table. Under the *Loaded* condition, after hearing the auditory cue the participant first lifted their arm to the horizontal (90° SABD) level, then performed the open or close task *Volitionally* or with *FES* for 6 additional seconds. A rest period of at least 30 s was provided between trials to minimize fatigue.

#### 2.3.2 Peripheral nerve anesthesia block

**Figure 2:**
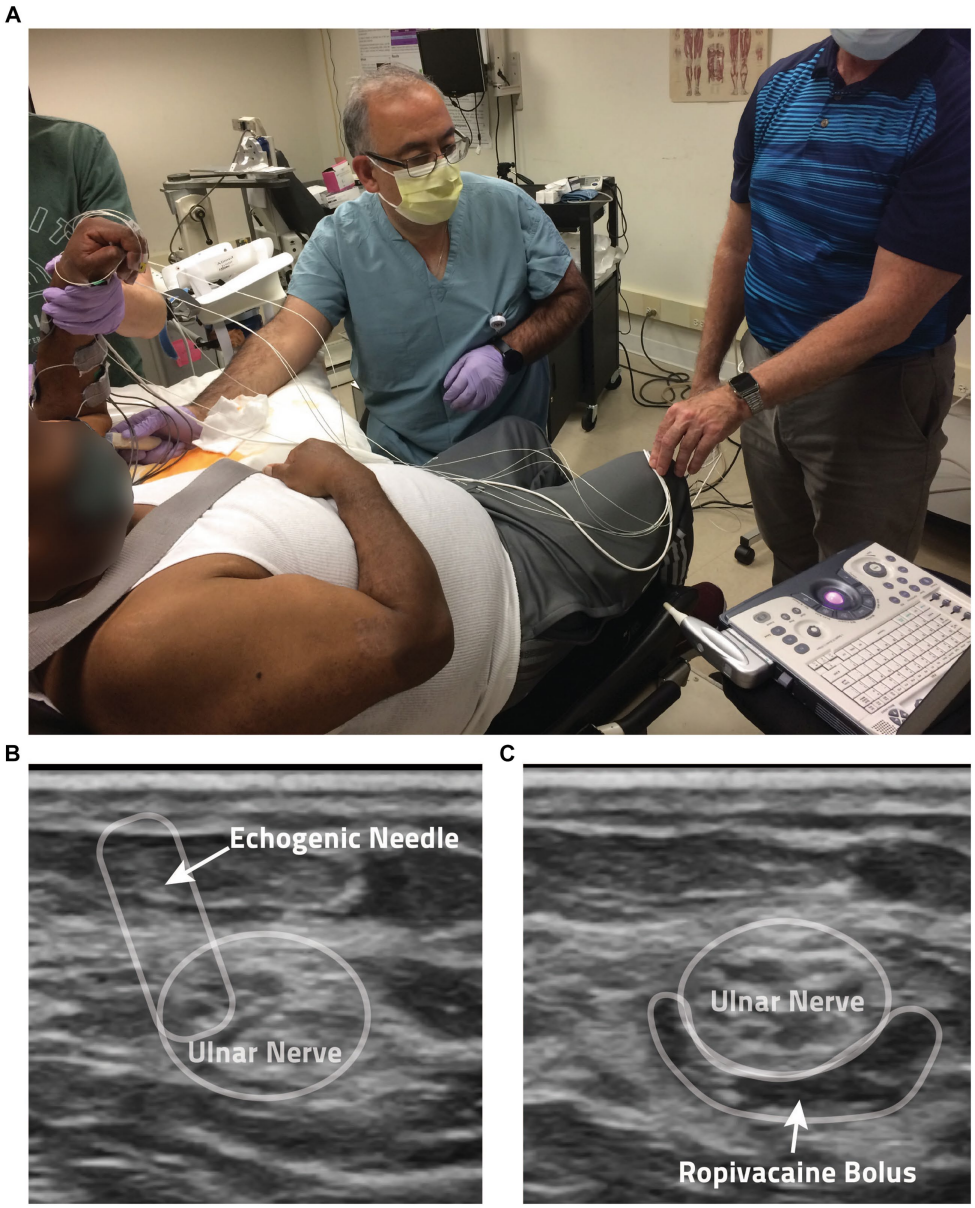
Anesthesia protocol. **(A)** Shown is a photo of the anesthesia application process. **(B)** Ultrasound guidance is used by the anesthesiologist in the administration of the local anesthesia ropivacaine. Shown here is the perineural injection method employed, with the echogenic needle noted by the arrow. **(C)** In this ultrasound image, you can see the anesthesia bolus surrounding a participant’s ulnar nerve following injection.

After the data collection for the *Unblocked* condition described above, the participant was prepped for applications of Ropivacaine to the ulnar and median nerves in the upper arm to induce a block of all wrist and finger flexors. After cleaning the skin with ChloraPrep (Becton, Dickinson and Company, Franklin Lakes, NJ), a trained anesthesiologist identified the position of each nerve via Ultrasound (GE LOGIQ e Ultrasound, 12L transducer, GE, Buc, France) and a SonoPlex echogenic nerve block needle (PAJUNK, Geisingen, Germany). Once a nerve was located, 5 mL of 0.5% Ropivacaine (Naropin, AstraZeneca, Wilmington, DE) was applied via perineural injection (see Figure 2). Throughout these injections, electrocardiogram, heart rate, and blood pressure were closely monitored (GE Carescape B105, GE Healthcare, Chicago, IL) for any adverse reactions.

#### 2.3.3 After anesthesia (*Blocked* condition)

After a 1.5 h rest period during which the block effect was allowed to plateau, the same data collection under all the various conditions described in the *Unblocked* condition was repeated for the *Blocked* condition.

### 2.4 Data collection

Data was recorded using a custom MATLAB program (Mathworks, Natick, MA) using API libraries from Pressure Profile Systems and Northern Digital. The flexion force was measured by the pressure sensor mat sampled at 16.5 Hz. The finger position was measured by the trakSTAR system sampled at 30 Hz.

### 2.5 Data analysis

#### 2.5.1 Outcome measures/metrics

**Figure 3:**
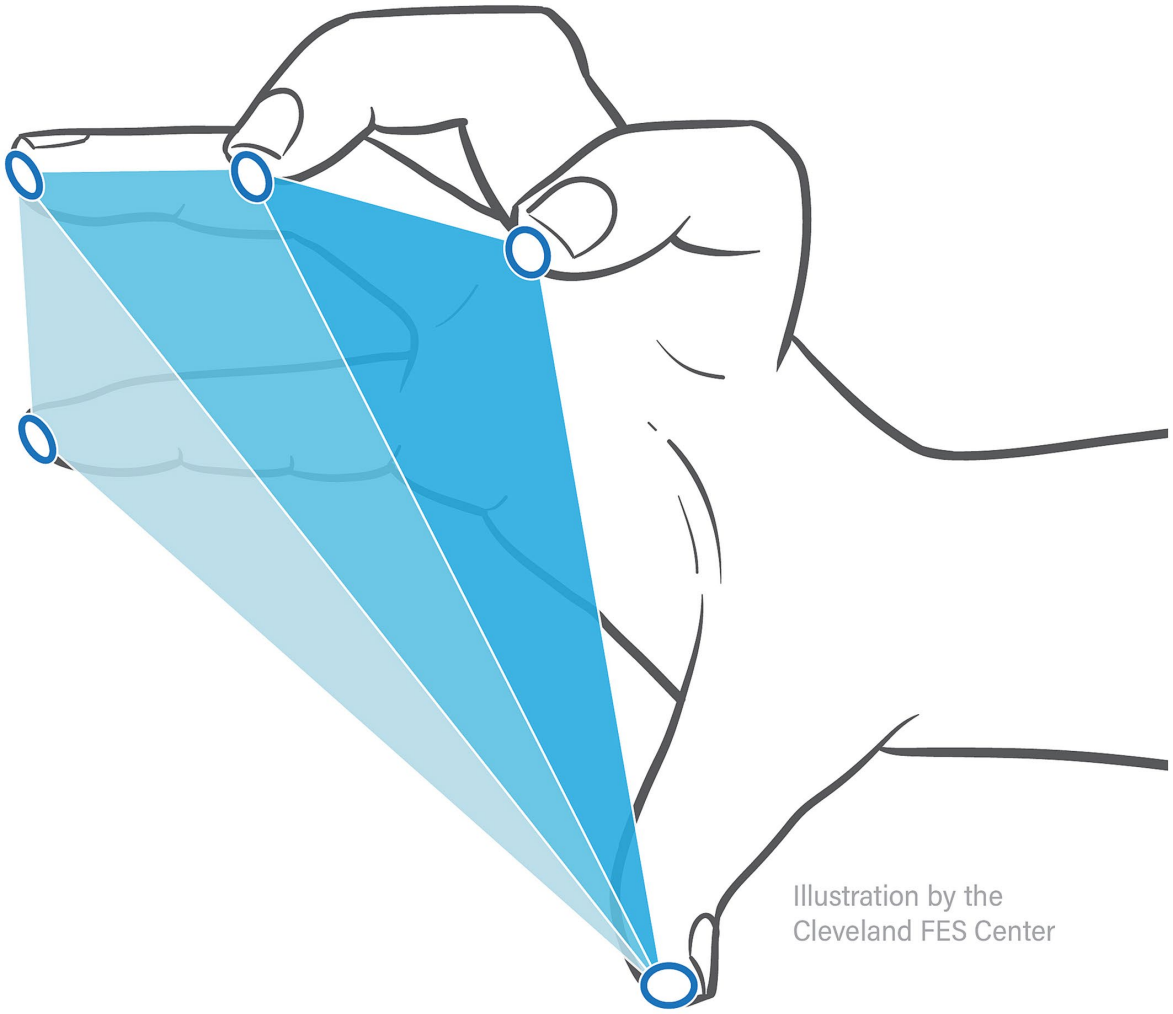
Hand pentagon area. Hand pentagon area, or HPA, is found using the trakSTAR sensor positions by calculating the surface area of three triangles made by the 5 fingertips: thumb-index-middle, thumb-middle-ring, and thumb-ring-pinky.

Hand pentagon area (HPA), shown to be an effective measure in evaluating hand opening ability (2), was used as the primary outcome measure when quantifying hand opening. As shown in Figure 3, this area (in mm^2^) was calculated as the sum of the surface area of three triangles formed by the participant’s fingertip sensor locations in 3D space: thumb-index-middle, thumb-middle-ring, and thumb-ring-pinky. The maximum HPA presented during each *Hand Opening* trial was calculated. The average of these max HPAs across trials of the same conditions was then normalized by each participant’s largest HPA (across all conditions), providing a 0–100% hand opening metric that could be readily compared across participants and between conditions.

To determine block success and gauge the impact of nerve block on FES function, the total grasp force generated by each participant’s fingers and wrist in pounds (lbs) was calculated from pressure mat data by multiplying the PSI value of each sensor by each sensor’s size (6 by 6 mm). The maximum presented grasp force during a *Hand Closing* trial was determined, ensemble-averaged across each participant’s trials within the same condition, and finally normalized by each participant’s largest *Volitional* grasp force.

**Figure 4:**
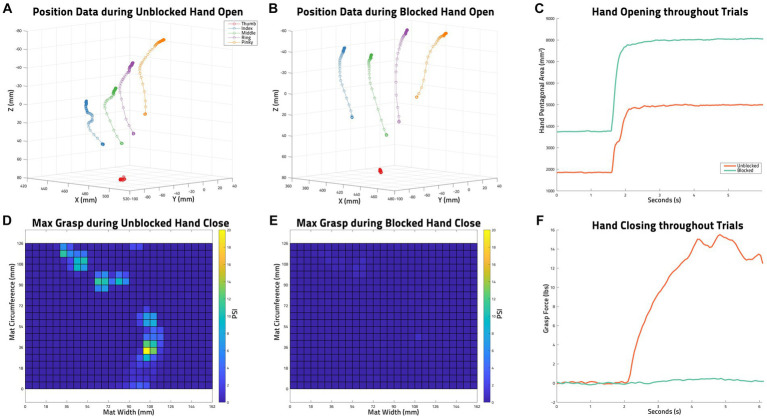
Example of Collected Data. **(A)** trakSTAR position data of all sensors during an unblocked hand opening task by S03: functional electrical stimulation (FES), unloaded, trial 3. **(B)** trakSTAR position data of all sensors during a blocked hand opening task by S03 (FES, unloaded, trial 2). **(C)** Hand pentagon area (HPA) as calculated from the sensor positions shown in **(A,B)** throughout the two mentioned trials. **(D)** The maximum grasp force measured with the PPS pressure mat during an unblocked volitional hand closing task by S03 (unloaded, trial 2). **(E)** The maximum grasp force measured with the PPS pressure mat during a blocked volitional hand closing task by s03 (unloaded, trial 1). **(F)** The grasp forces in lbs calculated from PPS pressure mat data (maxes of which are shown in **D,E**) throughout the two mentioned trials.

An example of the collected HPA of two *Hand Opening* trials (*Unblocked* and *Blocked* of the same conditions) and the grasp force values of two *Hand Closing* trials (*Unblocked* and *Blocked* of the same conditions) can be seen in Figure 4.

#### 2.5.2 Statistical analysis

At the individual level, paired *t*-tests were used per participant for *Volitionally*-driven Close and for *FES*-induced Close, separately, to verify that the nerve block significantly reduced grasp forces while not having any impact on FES behavior.

A linear mixed effects regression (LMER) model was then created to determine if observed significant differences in FES-induced grasp force followed a consistent trend dependent on the *Block* condition.

LMER models were also used to determine the impact of *Block* on hand-opening. Four models were made for the following conditions: *Volitional* and *Unloaded*, *Volitional* and *Loaded*, *FES*-driven and *Unloaded*, and *FES*-driven and *Loaded*. All data used in these models were normally distributed (Shapiro–Wilk test) so as to satisfy LMER assumptions.

Statistical significance was set at *p* < 0.05. All statistical analyses were performed in R (The R Foundation, Indianapolis, IN).

## 3 Results

### 3.1 Determining block success

**Figure 5:**
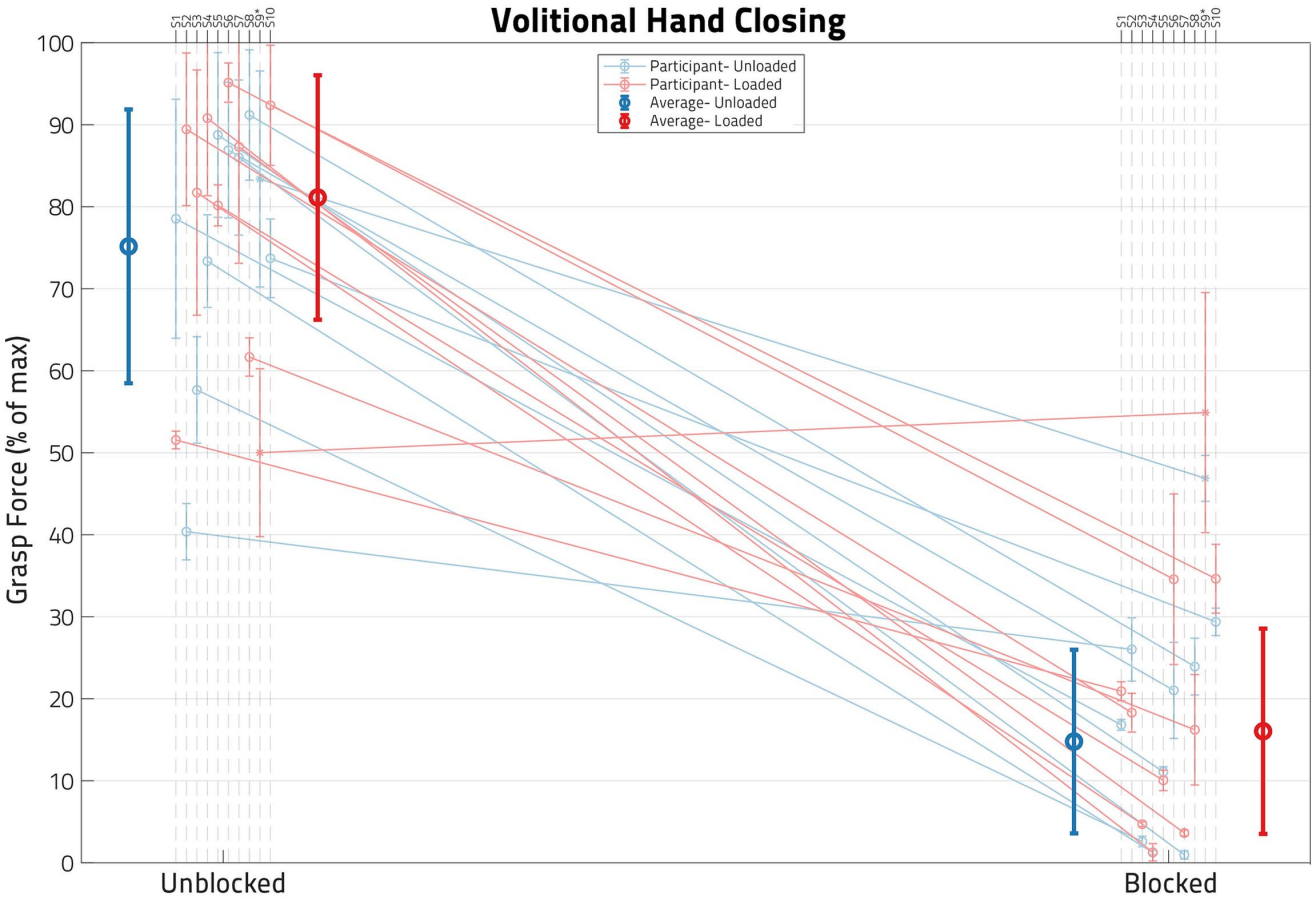
Impact of block on volitional hand closing. Each dot represents the normalized mean grasp force for a set of load and block conditions, with the error bars denoting standard deviation. Unblocked means are on the left and blocked means on the right. The blue lines represent *Unloaded* shoulder trials and the red lines represent *Loaded* shoulder trials. The blue and red circles on the left are the cross-participant averages for the *Unblocked* condition, while the corresponding circles on the right are for the *Blocked* condition. Note that, following block, grasp forces are reduced in most participants, verified by a paired *t*-test (*p* < 0.05). Only one participant, S09, failed to demonstrate statistical difference between pre- and post-nerve Block.

The ability of the anesthesia block of the median and ulnar nerves to reduce hand flexor muscle forces is illustrated in Figure 5, which shows flexion forces before and after the block for each of the participants in this study. To ensure a successful block, grasping forces were measured during (1) voluntary hand closing while the arm was supported (*Unloaded*) and (2) simultaneous voluntary and synergy-driven hand closing from lifting the arm against a load (*Loaded*). For all participants except one (S09), the anesthesia block produced large decreases (average 75%) in grasp force; S09 is denoted with an asterisk in all figures for this reason.

A paired *t*-test per participant (using *all* of their *Volitional* Close trials, both *Unloaded* and *Loaded*) indicated a significant (*p* < 0.05) drop in grasp force following the nerve block in all participants except S09 (whose means are represented by an asterisk in Figure 5 instead of a circle). Table 2 shows both the grasp forces of each participant and the percent drop in *Volitional* grasp force due to anesthesia nerve block per participant. Participant S09 had a much lower grasp force than any of the other participants, and the relative decrease (36.5%) in grasp force was significantly lower than the other participants. A successful nerve block was defined as a drop of at least 50% *Volitional* grasp forces following the application of anesthesia (4). Thus, S09 was not included in any subsequent statistical analyses.

**Table 2:** Maximum possible block impact per participant.

ID	FMA UE (/66)	Maximum vol grasp (lbs)	%Vol grasp drop by block	% open increase by block	% Vol open increase by block	% FES open increase by block
S01	33	47.47	61.71	47.45	3.87	47.43
S02	18	27.76	71.14	35.40	15.36	1.36
S03	29	25.10	79.12	77.96	41.20	36.94
S04	24	32.70	89.55	68.75	4.43	50.57
S05	20	55.33	78.70	3.41	−2.04	0.07
S06	20	6.92	74.10	71.79	65.12	19.66
S07	43	29.73	86.35	−6.93	−7.96	−13.79
S08	47	34.99	74.96	68.95	67.97	66.92
S09	13	3.18	36.50	60.26	32.21	23.15
S10	47	18.58	62.99	49.60	49.61	32.09

All data within the four % columns were calculated from the means of each set of conditions (Open/Close, *Vol*/*FES*, *Unloaded*/*Loaded*, *Unblocked*/*Blocked*), taking the largest mean value from applicable conditions and subtracting from it the smallest mean value from the unblocked corollaries. “Vol” stands for “Volitional,” FES stands for Functional Electrical Stimulation, and FMA UE stands for Fugl–Meyer assessment of the upper extremity.

### 3.2 FES behavior post nerve block

**Figure 6:**
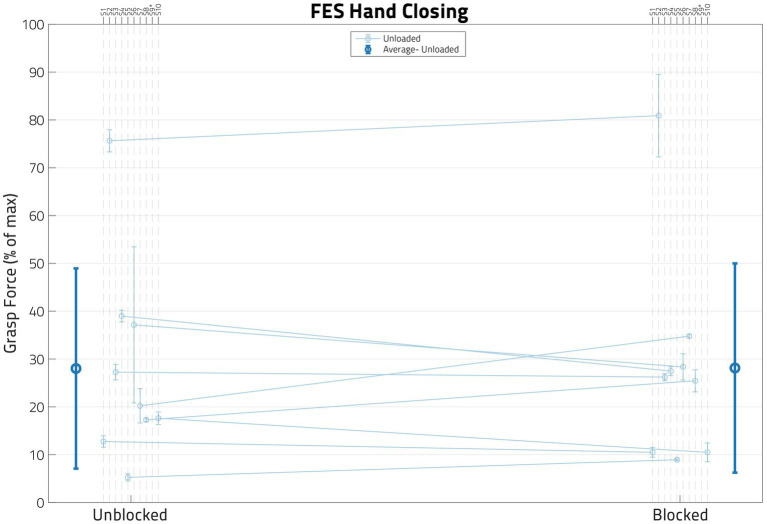
Impact of block on FES behavior. In this figure, each dot represents the normalized mean functional electrical stimulation (or FES) induced grasp force from the trials for one participant, with the error bars denoting standard deviations. While paired *t*-tests showed that these two populations were significantly different in some participants, a linear mixed effects regression (LMER) model found that there was no global trend. S09’s data is not shown in this figure as their FES grasp forces surpassed the volitional grasp force 0–100% range.

The impact of the anesthesia block on the hand flexion forces elicited by FES (applied at the forearm, below the elbow) is illustrated in Figure 6. The mean normalized FES-induced grasp forces before and after the nerve blocks are shown for the participants, along with their accompanying standard deviations. The average change in normalized FES-elicited grasp forces across participants before and after the block was very small—it is shown by the darker line, along with its associated standard deviation.

A paired *t*-test per participant using all *FES Unloaded* trial data indicated that the nerve block did change the FES-induced grasping force (*p* < 0.05) for 6 out of 9 eligible participants. Furthermore, an LMER model that included only *Block* as the Fixed Effect and *Participant* as the Random Effect was used to determine whether nerve block has any effect on FES-driven hand closing forces under the *Unloaded* condition (Table 3). The model did not find *Block* statistically significant (*p* ≫ 0.05).

**Table 3:** LMER results.

**FES unloaded hand closing LMER**				
Normalized Grasp ~1 + Block + (1|Subject: Trial) + (1|Block: Subject)				
*Term*	*Estimate*	*95% Confidence intervals*		*p*-*value*
Block	0.2994	−19.25	19.83	0.97652
**Hand Opening LMERs**				
Normalized opening ~1 + Block + (1|Subject: Trial) + (1|Block: Subject)				
**Volitional and Unloaded**				
*Term*	*Estimate*	*95% Confidence intervals*		*p*-*value*
Block	11.838	−8.49	32.17	0.273
**Volitional and Loaded**				
*Term*	*Estimate*	*95% Confidence intervals*		*p*-*value*
Block	12.538	−8.02	33.10	0.252
**FES and Unloaded**				
*Term*	*Estimate*	*95% Confidence intervals*		*p*-*value*
Block	3.129	−7.88	14.15	0.587
**FES and Loaded** ^*^				
*Term*	*Estimate*	*95% Confidence intervals*		*p*-*value*
Block	19.431	1.56	37.30	0.0499^*^

Shown below are the linear mixed effect regression (LMER) models created for the functional electrical stimulation (FES) hand closing and all hand opening results. While individual *t*-test results for FES hand closing found significant differences pre/post *Block* for 6 of the 9 eligible participants, the LMER model did not find *Block* to be significant, and the coefficient estimate is near 0, suggesting no consistent trend. Regarding hand opening LMER data, all four models provided a positive coefficient for *Block*, but only in the *FES* + *Load* case was the difference found to be significant.

### 3.3 Hand opening

**Figure 7:**
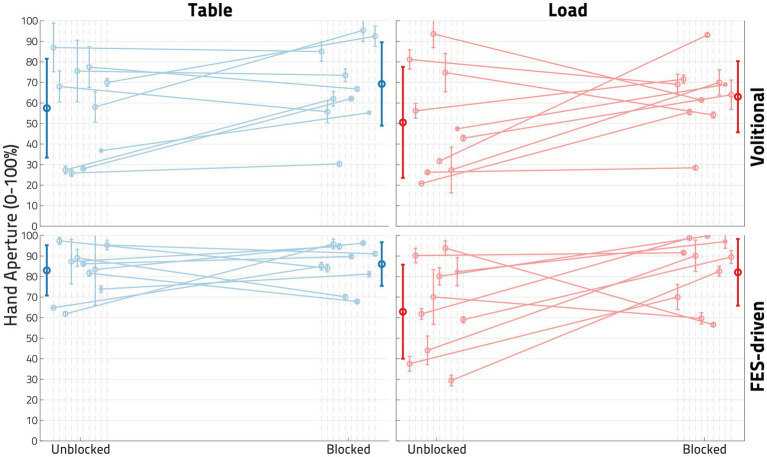
Impact of block on hand opening. In each plot, each dot represents the normalized mean hand pentagon area (HPA) from the trials of one participant in the denoted conditions, with the error bars denoting standard deviations. The left column of plots showcases the *Unloaded* (or table) condition, while the right represents the *Loaded* condition. The top row shows the *Volitional* condition, while the bottom row shows the functional electrical stimulation (*FES*)-driven condition. Four linear mixed effects regression (LMER) models were created for the four plots shown. In the bottom right plot-the FES-driven w/load condition- the *Block* term was found to be statistically significant (*p* < 0.05).

Figure 7 shows the hand opening expressed as hand pentagon area (HPA) of all 10 participants, normalized per participant to their largest observed HPA. While most participants demonstrated an increase in hand opening following block for most conditions, this behavior was not ubiquitous in our 9-person sample (see S05 and S07 in Figure 7 and Table 2). The block had its largest and most consistent effect for FES-elicited contractions while supporting a load at the shoulder.

Four LMER models were created to determine the impact of *Block* on hand opening under the conditions of interest (*Loaded* vs. *Unloaded* and *Volitional* vs. *FES*). While all four models found that *Block* had, when viewed across the 9 participants included, a positive impact on hand opening, only in the *FES*-driven and shoulder-*Loaded* condition was the effect found to be significant (*p* < 0.05). In this model, the coefficient of block was found to be 19.431, or a roughly 20% increase in hand opening following the application of the nerve block.

To verify that the nerve block reduced the impact of shoulder loading on FES hand opening, we performed an additional one-tailed paired *t*-test on the change in FES hand opening brought on by loading, shown in Table 4. The drop in FES hand opening induced by *Load* was found to be significantly less in the *Blocked* case than in the *Unblocked* case (*p* = 0.02198).

**Table 4:** Load impact on FES opening, unblocked vs. blocked.

ID	Load delta, unblocked	Load delta, blocked
S01	−27.2609	−14.9751
S02	−7.14372	7.469255
S03	−0.02571	2.925072
S04	−43.2273	−4.49613
S05	−19.0013	−10.5008
S06	−6.0757	10.02314
S07	12.22537	−11.2919
S08	−54.0182	−13.7437
^*^S09	8.474343	15.8219
S10	−36.3131	−1.59081

Load Delta in this table is defined as the average Functional Electrical Stimulation (FES) -driven Hand Opening per participant (normalized by each participant’s maximum Hand Pentagon Area, or HPA) in the Unloaded condition minus that of the Loaded condition. These values were calculated per participant, both before nerve block (middle column) and after (rightmost column). FES Hand Opening has been known to suffer severely following the addition of a load at the shoulder (as shown by large negative deltas), but the nerve block has reduced this issue for most participants; a one-tailed paired T-Test (*excluding S09) found that nerve block significantly reduced the impact of Load on FES-driven Hand Opening.

## 4 Discussion

### 4.1 Summary of findings and previous research

Our results demonstrated that Ropivacaine injection in the median and ulnar nerves induced a block of hand grasp in 9 out of 10 participants with moderate to severe stroke, averaging 75% of their maximum hand grasp force. Furthermore, we demonstrated that FES was able to produce flexion forces distal of the flexor nerve block sites. The effectiveness of FES distal to the nerve block injection sites is critical as it allows for the possibility of performing a functional hand task with FES assistance following a block. Most importantly, this study has shown that blocking the median and ulnar nerves responsible for wrist and finger flexion *can* improve FES-assisted hand opening outcomes even during shoulder abduction loading conditions. Previous literature had demonstrated the potential of nerve block approaches to address abnormal passive and active torques at the first MCP joint (4). Our current study demonstrated the effect of nerve block on a more functionally relevant measurement of hand opening (HPA), and, for the first time, in reducing the detrimental impact of shoulder abduction loading induced flexion synergy on hand opening.

Assistive FES has been employed to improve hand opening in individuals with stroke (13), but its functionality has been significantly limited by flexion synergy expression. There have been multiple attempts to reduce this synergy expression to increase hand opening outcomes, such as by also utilizing FES for shoulder muscles to reduce synergy presentation (15), or designing arm support devices (44) such as the SaeboMAS. The combination of flexor nerve block and extensor FES shown here addresses these prior limitations with FES interventions and improves the feasibility of using modern FES approaches (14) to enhance hand function following stroke, even in the most severely impaired individuals with tremendous extensor weakness.

### 4.2 Study limitations and future work

#### 4.2.1 Sample size

The original power analysis (G*Power, Heinrich-Heine-Universität, Dusseldorf, Germany) for this proof-of-concept study used an estimated effect size and standard deviation based on work that used an alternate measure of load at the shoulder (see 4.2.2). While 9 participants were sufficient to establish statistical significance in one set of conditions, a larger sample size could provide greater clarity on the impact of nerve block across the wide level of impairment encompassed by our participants. Of note is that the impact of nerve block on *Volitional* hand opening was still quite staggering in some individuals (such as S06), suggesting that *some* individuals could already benefit tremendously from nerve block alone without any assistance from FES. Determining which individuals may see such outcomes depends not only on a better understanding of mechanisms (4.2.5) but also on a larger sample size.

#### 4.2.2 The effect of shoulder abduction loading

This study included trials with loading at the shoulder to evaluate the ability of nerve block to reduce the negative consequences of the flexion synergy on hand opening (2, 8). We studied two simple loading conditions: *Unloaded*, or when the participant was resting on a haptic table generated by the ACT^3D^, and *Loaded*, when the participant had to lift the full weight of their limb (100% limb weight, or 100%LW, as used in prior studies (41)). Future studies should more completely take into account the varying levels of participant impairment and shoulder strength so as to remove the variability introduced by relying upon participant limb weight. One such metric that better normalizes results between participants of varying levels of impairment, strength, and limb weight is percentage of maximum voluntary torque (MVT) expression at the shoulder (45, 46); using metrics such as these should reduce the variability in resulting data and may provide greater insight into the variation in efficacy of nerve block in hand opening across different levels of stroke impairment.

#### 4.2.3 Block success and impairment levels

Block success was determined by measuring the drop in volitional hand closing forces following the administration of ropivacaine using a within-subject comparison *t*-test. Nine out of 10 participants indeed showed large drops in volitional hand closing forces, averaging 75%. Some participants had particularly low volitional grasp forces prior to the nerve block as compared to those generated by FES, which made determining block efficacy in these participants more difficult. We believe that S09’s failure to reach statistical significance and the 50% drop cutoff is due, in part, to the reduced dynamic range of the pressure mat at S09’s lower grasp force levels. Although ropivacaine did not result in significantly reduced hand closing in S09 (for which their data was excluded from further statistical analysis), we still observed a ~60% increase in hand opening from S09 following the nerve block, indicating that this block improved hand opening (S09’s data in the figures is denoted by an asterisk).

Regarding FES hand closing we observed minor, but statistically significant, variations in grasp forces before and after nerve block in 6 individuals (some increasing and others decreasing). The *Unblock* and *Block* FES Close cases have 2+ hours between them; many changes, such as fatigue and electrode site property changes, could occur during this period that can explain the change in grasp force. The statistical analysis based on the group data (see Table 3) did not show significant change in FES-induced flexion force. This result-that FES efficacy downstream of a nerve block site would perform similarly to the unblocked condition— was anticipated and supported by current understanding of the mechanisms of FES stimulation of muscle, but no prior scientific literature has technically demonstrated this. Now that this has been shown, FES assistance for flexors could arguably be applied alongside nerve block approaches in future interventions that might be unable to provide the required partial blocking or immediate on/off control of flexors necessary for an intervention useful for activities of daily living.

#### 4.2.4 Mechanical side effects of nerve block

Though the LMER models showed a significant increase in FES hand opening ability during Load across our 9 participants, a few participants (S05 and S07) exhibited a decrease in FES or Volitional hand opening following the nerve block (Figure 7 and Table 2). We have considered two possible explanations for this: Firstly, ropivacaine nerve blocks affect not only motor, but also sensory nerve fibers, and the impact of this loss of afferent information in the spinal motor neuron loop on antagonist (extensor) behavior is not entirely clear. Secondly, loss of intrinsic hand muscles has often impaired FES hand outcomes in Spinal Cord Injury interventions, resulting in “claw hand” (47). This presents as strong finger flexion at the second and third MCP joint, reducing the HPA and thereby the potential for grasp functionality. We had occasionally observed such presentations in some of our participants, but our HPA metric did not take the orientation of the sensor into account. Future work could potentially omit ulnar nerve block or include FES of intrinsic hand muscles. Using implanted FES electrodes would, in general, provide precise, selective activation of the hand muscles needed to provide a more normal hand grasp pattern (48).

#### 4.2.5 Disability mechanism contributions

Using electromyography (EMG) signals of relevant musculature as well as by calculating the purely flexion synergy-driven grasp forces exerted on the pressure mat during lifting, we next plan to analyze more directly the impact of the nerve block on certain known mechanisms of stroke disability. Of particular interest are the impacts of nerve block on the expression of hypertonia (tonic activation of wrist and finger flexors even while at rest), co-contraction (simultaneous activation of wrist and finger flexors and extensors during certain tasks (8)), and the expression of the flexion synergy (activation of wrist, finger, and elbow flexors proportional to the activity of shoulder abductors). This could help to explain why some participants improved in hand opening while others did not.

#### 4.2.6 Electrical nerve block

There are a variety of electrically driven nerve block approaches currently in development (33) that could perform a similar function as Ropivacaine did in this study. KiloHertz frequency alternating current in particular (KHFAC) could provide a means for user-controlled, on/off, instant, and reversible flexor nerve block. Some studies have also demonstrated the potential for partial blocks using KHFAC (49). Regardless of whether managed by KHFAC block alone, or combined with newer emerging DC block approaches (50), a temporary and instant reduction of flexor activity combined with FES-assisted extension could provide a permanent solution to functional losses at the hand in individuals with moderate to severe chronic stroke.

#### 4.2.7 Alternate hand ability metrics

More work needs to be done to evaluate the impact of a general increase in hand opening (as measured by HPA) on activities of daily living. The “clawhand” presentation we observed in some participants may limit functional gain for certain individuals. A possible means to better account for “clawhand” while measuring hand aperture could be a hand hexagon area (HHA), where an additional sensor on the center of the back of the hand could serve as a reference (ref) for four triangles: ref-thumb-index, ref-index-middle, ref-middle-ring, and ref-ring-pinky. Lastly, comparing HPA, HHA, or any other hand-opening metrics against functional tests such as box and blocks or clothespin task could provide greater insight into the true value of a combination nerve block and assistive FES approach.

## 5 Conclusion

Blocking undesirable and abnormal hand flexor contractions in individuals following hemiparetic stroke using local anesthesia of the median and ulnar nerves was shown to improve the ability of most individuals to open their hands using assistive functional electrical stimulation of the hand extensor muscles. These results indicate that controllable and deployable methods for blocking peripheral nerves, such as electrical block, may facilitate the deployment of better, proven FES methods for hand functional restoration for individuals with hemiparetic stroke.

## Data availability statement

The raw data supporting the conclusions of this article will be made available by the authors, without undue reservation, upon request.

## Ethics statement

The studies involving humans were approved by Northwestern University Institutional Review Board (IRB #STU00213403). The studies were conducted in accordance with the local legislation and institutional requirements. The participants provided their written informed consent to participate in this study. Written informed consent was obtained from the participants for the publication of any potentially identifiable images or data included in this article.

## Author contributions

HD: Conceptualization, Data curation, Formal analysis, Investigation, Methodology, Project administration, Software, Writing – original draft, Writing – review & editing, Resources. JY: Data curation, Formal analysis, Methodology, Project administration, Supervision, Writing – review & editing. JD: Investigation, Methodology, Writing – review & editing, Conceptualization, Funding acquisition, Project administration, Resources, Supervision. AN: Conceptualization, Methodology, Writing – review & editing, Investigation. RK: Conceptualization, Formal analysis, Funding acquisition, Investigation, Methodology, Project administration, Resources, Supervision, Writing – review & editing.

## Glossary of Acronyms

FES: Functional electrical stimulation
FMA UE: Fugl–Meyer assessment of the upper extremity
mL: Milliliter
IRB: Institutional review board
ACT^3D^: Arm coordination training 3D
SABD: Shoulder abduction
PSI: Pounds per square inch
FDS: Flexor digitorum superficialis
EDC: Extensor digitorum communis
μs: Microseconds
Hz: Hertz
ms: Milliseconds
API: Application programming interface
HPA: Hand pentagon area
mm^2^: Millimeters squared
lbs: Pounds
LMER: Linear mixed effect regression
ANOVA: Analysis of variance
Vol: Volitional
LW: Limb weight
MVT: Maximum voluntary torque
EMG: Electromyography
